# Patient safety when receiving telephone advice in primary care – a Swedish qualitative interview study

**DOI:** 10.1186/s12912-021-00796-9

**Published:** 2022-01-19

**Authors:** Karin Berntsson, Maria Eliasson, Linda Beckman

**Affiliations:** 1 Department of Health Sciences, Karlstad University, Karlstad, Sweden; 2 Department of Health Sciences, Public Health Science, Karlstad University, Karlstad, Sweden

**Keywords:** Communication, Content analysis, District nurse, Interview, Patient safety, Telephone advice

## Abstract

**Background:**

A lack of patient safety is a significant global public health challenge and is one of the leading causes of death and disability, entailing significant financial and economic costs. However, patient safety can be improved and patients can avoid being harmed if more knowledge could be gained about what it is that impacts patient safety. Patient safety when receiving telephone advice is an important issue given the increase in digitalization in healthcare services.

**Aim:**

The aim of this study was to explore district nurses’ (“telenurses”) experiences and perceptions of patient safety when providing health advice over the phone.

**Methods:**

Data collection was performed using semi-structured interviews and analyzed using qualitative content analysis. The participants (*n* = 12) were telnurses in primary care.

**Results:**

The theme “Being able to make the right decision” was formed based on two categories: “Communication” and “Assessment”. Through effective communication with the right conditions to make an assessment, the correct decision can be made when a patient calls, and the district nurse feels that their telephone advice is safe for the patient.

**Conclusions:**

Patient safety can be challenged when receiving telephone advice, particularly when they feel stressed due to organizational factors. There is a need to shift from the individual to the organization. Further, while computerized knowledge support generally results in safe decisions, there may also be problems. Hence, it is imperative to develop computerized knowledge support as a part of improved patient safety in telephone advice.

**Supplementary Information:**

The online version contains supplementary material available at 10.1186/s12912-021-00796-9.

## Introduction

Telephone advice is often a patient’s first contact with health care for help with their problems. Telephone advice service is increasing globally, and in a world characterized by an increasing proportion of older people and more immigrants with language difficulties [1], as well as increased digitalization in healthcare services [2], patient safety in telephone advice is an important issue. Telephone advice service is integral to modern health care and offers fast, low-cost, geographically unrestricted and anonymous access to health information and advice [3]. Sweden has a national helpline (1177) for health advice, which is a cooperation among all regions in the country, and a person can also call primary health care or private online health care services. No matter which service a person calls, a nurse (hereby called “telenurse”) usually answers.

The assessment process is facilitated by computerized knowledge support, an online decision-support system (DSS) which, for example, supplements knowledge in areas in which clinical knowledge is limited. Computerized knowledge support provides a greater level of certainty when investigating health issues and ensures better quality when offering advice over the phone [4]. The most common Swedish DSS is called the “RGS webb”. The purpose of this service is to help triage patients when they call for help and counseling. The RGS webb includes the patients’ medical information and documentation from the patient’s record [5]. Previous studies on DSS in both the USA [6] and Sweden found that using a DSS led to fewer mistakes and better triaging. However, according to a Swedish study by Holmström et al. [7], multiple factors impact the use of a DSS: extended working experience, lack of time (since a DSS includes larger volumes of text that takes a long time to read) and training in DSS. Sometimes a DSS did not fully correspond to the working situation and nurses needed to make their own decisions.

Although telephone advice has many benefits, it also has its limitations. To be able to “read between the lines” and understand a patient’s needs based on a conversation is an important part of the decision-making process because it is not possible to factorize body language and facial expressions into an assessment [8]. Some older people have difficulties using a telephone advice service and participating in such conversations. Thus, it is important that the telenurse communicates clearly and maintains focus throughout the conversation [1]. Lack of communication due to unstructured assessment techniques or technical problems [9, 10], linguistic difficulties or cultural differences, and that callers provide either too much or too little information [10], can lead to a lack of information. A Swedish qualitative study [11] on telenurses found that there is a risk of misjudgment when parents call on behalf of their child, because it may be more difficult for the nurse to create a holistic picture of the child’s problems. A holistic picture might be obtained by being responsive, asking the right questions and reading between the lines [11, 12]. The work of telephone advisers can be perceived as being both positive and stimulating, but telenurses are also in a vulnerable position in which their advice must be correct and provide adequate help for patients [13]. Ström, Marklund and Hildingh [13] argue that such work requires the responding nurse to be continuously up to date on scientifically proven knowledge that is also credible.

According to Kaminsky, Rosenqvist and Holmström [14], a nurse’s task when offering telephone advice is to assess and refer patients, as well as establish the level of triage based on a patient’s problem, as well as support and empower them. The outcome of a call depends on the communication between patient and nurse. In a study by Gustafsson, Wälivaara and Gabrielsson [15], patient satisfaction with telephone nursing was about whether the nurse was *calm*, the *clarity* of the conversation (including distinct, concrete and practical advice on what to do and what to observe) as well as the nurse’s *competence* – including both knowledge and caring skills [15]. Nurses must also determine whether it is possible and if there are any prerequisites regarding the patient’s ability to implement and follow the advice that they have been given [14]. An observational study of a county population in Sweden found that 77% of callers from 2014 to 2015 followed the recommendations given to them by telenurses [16]. There is, however, a balancing act for the nurse between being a carer and a “bouncer” [12]. Telenurses have been described as having a “special power” over their patients [17], or as being gatekeepers [18] i.e. they decide which patients will qualify for an appointment.

### Patient safety

In Sweden, as well as internationally, patient safety in health care has been a prioritized issue [19] and further research is warranted [20]. Patient safety has been defined by the National Board of Health and Welfare in Sweden as “protection from care injury” and patient safety work as “work aimed at enhancing patient safety by analyzing, determining and obviating the cause of risk, as well as adverse and negative events” [21]. The World Health Organization (WHO) ([20] p.5) uses a more latent and holistic definition of patient safety: “A framework of organized activities that creates cultures, processes, procedures, behaviors, technologies and environments in health care that consistently and sustainably lower risks, reduce the occurrence of avoidable harm, make errors less likely and reduce the impact of harm when it does occur.” Boström, Nordström and Wilde-Larsson [22] argue that awareness and knowledge of patient safe care have increased in health care, even though the development in patient safety has found it difficult to keep up with other medical advances, which constantly creates new challenges for caregivers to be able to offer patient safe care. When nurses or other healthcare professionals collect information and communicate over the phone, some parts of the conversation are particularly critical, and this is where mistakes can be made. Collecting information and communicating with patients are key to telephone advice. Thus, there are different ways to achieve safe telephone advice. A Swedish study [23] that analyzed 121 phone calls to primary health care found that safe telephone advice was impacted by multiple factors, for example, the surrounding environment, how the healthcare unit was organized, and the patient’s personality. According to Röing, Rosenkvist and Holmström [23], the clearest threat to patient safety in telephone advice was that nurses missed something important when collecting information.

Support for nurses advising over the phone is designed to increase patient safety but nurses have sometimes regarded it as an obstacle to communication [24]. A literature review [25] found that the transition from physical contact with a patient to telephone advice requires effective collaboration between professionals in the workplace and reliable knowledge support in order to gain the patient’s confidence and achieve patient safety.

Collecting information and communication are important aspects of telephone advice, meaning that patient safe telephone advice is an important issue. Although telephone advice is widely used in both Swedish and international primary care, there is a gap in the field of telephone advice and the elements that influence the advice given by nurses over the phone [19, 20, 22]. According to the WHO [20], a lack of patient safety is a global public health issue and one of the leading causes of death and disability, entailing great financial and economic costs. Serious incidents relating to patients can cause healthcare workers psychological distress and feelings of guilt. However, patient safety can be improved and patients can avoid being harmed if more knowledge could be gained about what it is that impacts patient safety.

## Aim

The aim of this study was to explore district nurses’ experiences and perceptions of patient safety when providing health advice over the phone.

## Methods

We adopted a qualitative and exploratory approach using semi-structured interviews that were analyzed using inductive content analysis to gain insight into the perceptions of district nurses regarding patient safety when they provide telephone advice.

### Participants and settings

A district nurse is a specialist nurse with a broad range of skills. According to the Swedish Nursing Association [26], a district nurse’s skills involve professional activities in multiple sectors with an emphasis on primary care, municipal health care, child health care and school health care. The basis of a district nurse’s work is to support people of all ages using a health-promoting approach.

First, the operations managers of health centers approved the interview study before the district nurses were interviewed. The selected district nurses (*n* = 12) worked at two health centers in Sweden at which the first and second authors worked. We, therefore, chose to use convenience sampling [27]. All 12 district nurses agreed to participate in the study. The participants at each health center were professionally acquainted. At the selected health centers, the district nurses used around 6 to 7 min for any phone calls that required documentation. One health center was located in a sparsely populated area far from the nearest hospital, while the other health center was located in a medium-sized town with a hospital nearby. Only women worked at the health centers, meaning that only women were interviewed. The participants had worked as district nurse for 1 to 35 years. The inclusion criteria were that the participants must have worked at a health center for more than 1 year and work as a telenurse. We did not include new employees because we wanted to highlight patient safety based on a district nurse’s level of knowledge after the training and professional experience.

### Data collection

Data collection took place in September 2019 using semi-structured interviews (See Additional file 1 for the interview questions). Information letters were sent to the participants in advance of the interview, as well as when the interview took place, that participation was voluntary and that they could withdraw from the study at any time. The interviews were recorded and transcribed verbatim. According to Graneheim and Lundman [28], it is important to note body language and sounds such as laughter and sighs, if spoken words are to be interpreted correctly. Graneheim and Lundman [28] also point out that qualitative content analysis constitutes a form of dynamic communication between researchers and participants that requires a safe interview setting and trust between the researcher and participant. The district nurses could choose where they wanted to conduct the interviews. Some interviews were conducted during work hours in a quiet location in the workplace, while other interviews were conducted in the participant’s home. Each interview lasted around 30 min. After conducting 12 interviews, nothing new emerged in the conversations and we considered data collection to be completed.

### Ethical considerations

This study was approved by the local Ethics Committee at Karlstad University (no: 2019/760). Since the potential participants worked at the same workplace as the first and second authors, it was important to be sensitive to the fact that the nurses might feel that they were being coerced into participating. In order to mitigate this, the district nurses were informed that their participation was voluntary and that they were at liberty to withdraw from the study at any time without giving a reason.

### Data analysis

The transcribed interviews were read several times and compared through reflection and discussion between the first and second authors. The analysis was conducted in accordance with Graneheim and Lundman [28]. Meaning-bearing units were developed from the collected data by writing down all the responses that were appropriate to our purpose, i.e. the district nurses’ perceptions of patient-safe telephone advice. The meaning-bearing units were compared and given codes, and the codes were then categorized into two subcategories under each category. An overarching theme gradually emerged. Table 1 shows the process of data analysis.

**Table 1:** Example of the analysis process

Meaning-bearing units	Code	Subcategories	Category	Theme
It is important to make sure that you talk to the right person	Professional approach	Understanding	Communication	Being able to make the right decision
Some people make a mountain out of a molehill while others do the opposite	Being forced to read between the lines	Risk of misunderstanding		
RGS is useful for identifying symptoms and suitable treatment [for nurses]	Support	Prerequisites	Assessment	
Patients with mental illnesses and patients with chronic diseases need more time	Patients with serious health assessments	Obstacles		

### Reflexivity

The first and second authors are registered district nurses with some experience of providing telephone advice. The third author is an Associate Professor of Public Health Science. The backgrounds of the first and second authors might have influenced the results because of their pre-understanding standing of the participants’ work. However, the third author was involved in the analytical process, thereby increasing its objectivity.

## Results

The theme “Being able to make the right decision” was formed based on two categories: “Communication” and “Assessment”. Through effective communication, and good conditions to make an assessment, the right decision can be made when a patient calls, and the district nurse feels that telephone advice is safe for the patient.

### Communication

Clear communication between nurse and patient is crucial for the nurse to understand the problem that the patient is calling about. When there is a lack of communication, telephone advice is not safe for the patient.

#### Understanding the patient’s problems

It was important for the nurses to gain an overall picture of patients and their problems. For example, this could be achieved by using the patient’s medical records during the conversation to get some background, by listening to the patient, then asking additional questions about any inconsistencies, and by considering the bigger picture. The participants stated that active listening was important for understanding patients accurately by listening to what they are saying and then asking relevant follow-up questions. Active listening is about allowing patients to speak to the point, taking the time to focus on one patient at a time and allowing them to talk at their own pace so as not to unsettle them. One nurse said: *It is important to let the caller speak to the point and wait for any hidden thoughts to emerge when a conversation seems to start with a minor problem but ends up as something that is much more serious* (telenurse 10). For communication to work and telephone advice to be safe for the patient, the participants talked about the importance of a professional approach on the part of telenursing. This was achieved by ensuring that the nurse was speaking to the right person, that consent had been given if a family member had made the call, and that there was confidentiality, i.e. no outsider could overhear the conversation. Being clear was crucial for achieving effective communication and good understanding. The conversation aims to obtain a thorough understanding of the problem and a clear description of the symptoms. Then the telenurse must provide a clear answer. The participants provided examples of ways of being clear during such conversations, for example, repeating the information when it was being collected and also summarizing the most important aspects of the conversation before ending the call. *Different people explain their problems in different ways, and that’s an issue* (telenurse 1).

#### Risk of misunderstanding

When telenurses cannot see a patient and when there is a lack of communication, they can sometimes be forced to read between the lines. The participants perceived this as particularly difficult when they attempted to obtain the information that was necessaryfor understanding a problem, and a family member or friend had called on behalf of a patient. There is an increased risk of misunderstanding in such situations: *When talking to parents and their children, many questions are difficult to answer over the phone. So you need to see the child in person* (telenurse 3). It also emerged that patients sometimes had difficulty explaining what was wrong with them, i.e., some patients understated while other patients exaggerated their problems. The same problem could be described in many different ways by different patients. The participants also stated that many patients did not tell them everything that was relevant and might ignore problems, while other patients exaggerated their problems to receive faster help. *Some people make a mountain out of a molehill while others do the opposite* (telenurse 3). Language deficits in telephone interviews are a common cause of misunderstanding whereby someone stated that it is difficult to fully understand a situation when they do not speak the same language. It is difficult for a person to describe the problem they are searching for when their Swedish vocabulary is limited and the nurse is uncertain about whether they have made the right decision. The participants were worried about missing something important and explained that it was very difficult to talk to a family member whose Swedish was poor. In such cases, the participants suggested that telephone advice should take place in the patient’s language, if possible, to ensure that the advice was safe for the patient.

### Assessment

The participants stated that some factors contribute to a patient safe assessment while other factors make the assessment more difficult. In the study, these factors formed the subcategories of *prerequisites* and *obstacles.*

#### Prerequisites

The participants highlighted that experience was the most significant prerequisite. Experience contributes to a sense of security, and most problems presented by patients can benefit from knowledge and experience. *The more years you work, the easier it becomes* (telenurse 3). The participants believed that experience is more beneficial than training. However, district nurse specialist training is important and helps to ensure patient safe assessments. The participants also stated that they had not received sufficient training for offering telephone advice. However, they could update their knowledge and obtain tools to use in the telephone advice service. The RGS online support system was available at both locations at which the participants work and was described as a tool that would make phone advice patient proof, even if used in varying degrees. The web-based RGS support system aimed to ensure that the right questions were asked and that nothing was missed*. I use and follow the RGS, and by using it I can assess the degree of seriousness* (telenurse 2). Difficulty in using the RGS web-based support system could occur when a patient searched for multiple symptoms at the same time. The participants also stated that it was important to listen to what the patient says when searching in the RGS system, and to check the patient’s medical records. In addition to RGS, the national helpline 1177, Google, advisory books, and similar, were described as constituting different sources of knowledge and guidance for advising difficult patients.

When it was not possible to make an assessment directly over the phone, the participants said that they sometimes booked a physical appointment with the patient before an assessment was made. Web cameras was also being considered as an option in telephone advice as a way of ensuring patient safety. The participants regarded video calls as being more patient safe since it is possible to see the patient.

*If I'm going to tell it as it is*, *most of my conversations usually end up in an appointment, so the patient gets to visit us. I want to see the patient, sense how they’re feeling, make eye contact, be able to talk in peace and quiet, as well as do things that I can't do over the phone* (telenurse 12).

One option is to return to the patient after consulting with colleagues, which means that teamwork was also an important part of being able to make an accurate assessment. Having the option to seek help from physicians and other nurses helps the participants feel confident when making an assessment. In this respect, the participants described a good working climate as being an important prerequisite. This also included the physical surroundings: *Patient safety is increased when you minimize the distractions around the telenurse*, *such as people walking through the room and other distractions* (telenurse 1).

#### Obstacles

Advising over the phone was described as being challenging, regardless of how many years the telenurse had worked in the profession. When making an assessment during a telephone consultation, the participants stated that they felt that they needed to overcome various obstacles. Certain categories of patients were seen as particularly difficult to assess over the phone. These patients were identified as children and people with mental illnesses. Children were perceived as particularly difficult to assess because their parents often find it difficult to both assess and explain their child’s needs. The obstacles to assessing mental illness were identified as being time related. Such conversations often take a long time, even though each call is supposed to be limited to 6 to 7 min. *Patients with mental illness are a difficult group. Did I get the right answers when I asked my questions?* (telenurse 3). Most participants emphasized time constraints as being an obstacle. One nurse described having to *“Chase” those 6 min* (telenurse 5). Having to conclude all calls within 6 min to avoid falling behind was perceived as a potential source of stress, while the participants also emphasized that if they had more time, it would mean that they would be more likely to provide safer phone advice. The participants also stated that a lack of time also increased their workload, as well as the risk of becoming unfocused and incorrectly assessing a patient. *A prerequisite for being able to listen actively and make the right decisions is that you do not feel too stressed. It’s important to have a manageable reasonable workload.* (telenurse 2). Long shifts on the phone increase the risk of losing focus and providing adequate advice. An additional factor identified by the participants was insufficient knowledge and, in this context, ongoing training was highlighted as being the solution. *No matter how experienced you are, there will always be difficult cases* (telenurse 3). The lack of available appointments during a phone call was also described as a challenge when providing telephone advice, particularly for doctor’s appointments. The participants stated that in the event of uncertainty, they preferred booking an appointment once too often. When no appointments were available, they felt that they had to be more stringent regarding triaging in terms of which patients could wait and which patients could receive an appointment on the same day.

## Discussion

The results demonstrated that communication and the assessment of a patient’s problems were crucial in ensuring patient safe telephone advice. During a conversation, understanding a patient’s needs made the conversation more rewarding, while the risk of misunderstandings as a result of linguistic confusion, for example, led to communication difficulties. Providing an assessment over the phone was facilitated by the nurse’s experience and knowledge and was often hindered by external factors such as stress and the number of available appointments if the patient needed to see a doctor. Communication and assessment were two fundamental components that interacted simultaneously when nurses had to make a decision over the phone. Most of our results can be confirmed in previous literature. Nevertheless, these are still important findings as it suggests that such aspects still are a threat to patient safety when giving advice over the phone.

Our results show the importance of having an overview of the situation and listening actively during a telephone advice session. These are prerequisites for understanding a patient’s problems, in line with Purc-Stephenson and Thrasher [12]. It was also found to be important that nurses establish safe and positive contact with patients, without attempting to steer the conversation and without interrupting the patient [29]. However, it has been shown that establishing a positive interaction with callers can be problematic. Yliluoma and Palonen [30] found that disruptive background noises, agitated callers and other communication problems, as well as service system failures, challenged telephone interactions. Communication skills might be the most important skill for healthcare workers to acquire and are the basis of a professional approach, i.e., that professionals are guided by the patients’ needs and not their own feelings and needs. Empathy has been regarded as the basis of communication [31] and previous research has shown that health professionals who show a high level of empathy are more effective [32]. Although our participants did not explicitly use the word *empathy*, it was obvious that this was something they felt and made use of, as one participant stated: *I want to see the patient and sense how they are feeling.* However, many factors have been shown to negatively impact empathy [32], one of the most predominant ones being stress caused by lack of time, which can result in reduced patient safety [33] and which was frequently highlighted in our interviews. While empathy is more of an individual trait, it is the responsibility of the organization to mitigate stressful sources [34]. According to Reason [35], adopting a personal approach rather than a systemic approach (that focuses on working conditions and attempts to build defenses in order to avoid errors), delays the development of patient safety. By reducing complexity, optimizing information processing, using automation and constraints, as well as mitigating the unwanted effects of organizational change, patient safety can be improved [36]. These aspects could potentially be improved by the DSS because it simplifies the work of telenurses [37].

There is a risk that the patient’s right to self-determination (autonomy) could conflict with the nurse’s desire to do what is best for the patient, as the nurse has a responsibility to advocate for the patient [38]. One participant stated that patient safe telephone advice sometimes involves going against the patient. In other words, telephone advice is not always about being nice. However, the telephone advisers should view patients as a resource and support and develop patients’ health awareness [29, 39]. Bird [39] argues that the nurses’ role is complex, and is based on empowerment rather than advocacy and, according to Hyland [40], patient autonomy is not always compatible with the nurse’s role of advocacy.

In line with Barbosa et al. [4], we also found that one of the main challenges is difficulty in understanding patients and their problems when you cannot see them in person. Patient safety is entirely dependent on the quality of the communication process [4, 41]. However, as Pettinari and Jessopp [41] point out, there are also benefits. For example, patients may be uncomfortable talking about certain symptoms. Thus, describing their symptoms over the phone may be easier. Communication problems, such as the risk of misunderstandings, were identified when telephone advice involved a third party, e.g., when a parent called on behalf of their child [42]. Another problem was the lack of appointments and how this situation complicates telephone advice. When no appointments are available, the risk of feeling like a “bouncer” increases and may affect the decisions that are made [12, 17]. In line with Koivunen and Saranto [25], for example, the results of this study showed that the participants often made appointments so that they could see the patients in person, consult colleagues, such as doctors, and use counseling support when they were uncertain in order to ensure that their advice and decisions were correct. The participants stated that taking a chance was not an option when offering telephone advice. As confirmed in previous literature [4, 43], knowledge support is an important part of feeling confident about making an assessment, even if the nurses felt that their work experience was the most important prerequisite for making the right assessment when offering telephone advice. Feeling confident gives them the courage to make decisions.

### Limitations

Some limitations are worth noting that need to be considered in relation to the results. First, the interviews that took place in the participants’ homes were longer and involved more in-depth responses, compared to the interviews that were conducted in the workplace, which must be taken into account in terms of the credibility of the study. The participants interviewed in a home setting might want to describe themselves in a certain way, while an interview in the workplace might mean that the participants want to describe themselves in different ways, for example, that they want to come across as good employee or manager [44]. However, the interview analysis shows similar, and in some cases, identical responses from the different participants, regardless of the interview setting. Second, the interviewers worked at the same healthcare center as the participants. This convenience sample does limit the transferability of the results to the population as a whole [27]. It could further influence the results in terms of making the participants less likely to talk about more serious organizational issues. However, it could also make the participants more open and confidant with the interviewers. Third, only women participated in the study, which may have influenced the results.

## Conclusions

Telephone advice as a form of health care is an increasing phenomenon in primary care in both Sweden and other countries, and it is important to highlight how it can be conducted in a patient safe way. This study showed that telenurses still have many problems despite previous knowledge in this area. This could be because many of the issues related to patient safety, such as stress, are related to organizational and external factors, rather than individual factors. A well-functioning telephone advice service would benefit the entire healthcare system because it is usually the start of a patient’s care pathway. When telephone advice works properly, it is an effective way to triage patients to the right level of care, which is beneficial to both patients and healthcare professionals.

## Supplementary Information

**Additional file 1.**

## Data Availability

The datasets used and/or analyzed during the current study are available from the corresponding author on reasonable request.
