# Supplementary material for: Patient safety when receiving telephone advice in primary care – a Swedish qualitative interview study
Source: BMC Nurs. 2022 Jan 19;21:24. doi: 10.1186/s12912-021-00796-9 (PMC8767717; doi:10.1186/s12912-021-00796-9)
Supplement: Supplementary file 1 — Additional file 1. [file 12912_2021_796_MOESM1_ESM.docx]

**Interview guide with main questions.**

- How do you experience telephone advise?
- What does patient-safe telephone advise mean to you?
- Do you feel that telephone advise could be more patient-safe, and if so, how?
- When do you think telephone advise works and becomes patient safe?
- Do you use any knowledge support, and if so, how does this help you?
- Do you feel that you have sufficient conditions to conduct patient-safe telephone counseling?
